# Non-rigid point cloud registration based lung motion estimation using tangent-plane distance

**DOI:** 10.1371/journal.pone.0204492

**Published:** 2018-09-26

**Authors:** Fan Rao, Wen-long Li, Zhou-ping Yin

**Affiliations:** State Key Laboratory of Digital Manufacturing Equipment and Technology, School of Mechanical Science and Engineering, Huazhong University of Science and Technology, Wuhan, People’s Republic of China; North Shore Long Island Jewish Health System, UNITED STATES

## Abstract

Accurate estimation of motion field in respiration-correlated 4DCT images, is a precondition for the analysis of patient-specific breathing dynamics and subsequent image-supported treatment planning. However, the lung motion estimation often suffers from the sliding motion. In this paper, a novel lung motion method based on the non-rigid registration of point clouds is proposed, and the tangent-plane distance is used to represent the distance term, which describes the difference between two point clouds. Local affine transformation model is used to express the non-rigid deformation of the lung motion. The final objective function is expressed in the Frobenius norm formation, and matrix optimization scheme is carried out to find out the optimal transformation parameters that minimize the objective function. A key advantage of our proposed method is that it alleviates the requirement that the source point cloud and the reference point cloud should be in one-to-one corresponding relationship, and the requirement is difficult to be satisfied in practical application. Furthermore, the proposed method takes the sliding motion of the lung into consideration and improves the registration accuracy by reducing the constraint of the motion along the tangent direction. Non-rigid registration experiments are carried out to validate the performance of the proposed method using popi-model data. The results demonstrate that the proposed method outperforms the traditional method with about 20% accuracy increase.

## Introduction

Respiratory motion estimation is a vital problem in medical image processing [1,2]. The goal of the respiratory motion estimation is to acquire the time-sequenced motion fields along the lung surface. Usually, it is a precondition for many applications in medical image analysis, such as image-guided interventions, quantitative evaluation of the motion and generating dynamic numerical phantom data for assessment [1,3,4]. 4-D computed tomography (4DCT) technique has become highly popular for imaging respiratory motion [5–9]. It can generate a number of high-resolution volume images representing different phases in the respiratory cycle [10]. However, 4DCT images could not describe how tissues move and deform from one breathing phase to another breathing phase. They only provide static information of the patient's anatomy at different breathing phases. Algorithms to estimate the actual motion paths between these phases usually provide vector fields, which can be interpreted as motion fields. Then the motion fields form the basis of subsequent analysis steps and medical decisions. The estimated fields should therefore resemble breathing dynamics as accurately as possible. Accurate respiratory motion estimation is difficult due to the breathing mechanism. The breathing motion is arisen from the contraction of the diaphragmatic muscle area, and there exists severe sliding motion in the lateral areas of the lung. Many researchers indicate that the lung motion estimation suffers from the sliding motion [1,2,11], and a possible reason is that the sliding motion would plague the registration by the existence of local minima. Accurate respiratory motion estimation is important in radiation therapy of lung cancer. Usually lung motion fields are acquired before the radiation treatment, and the motion fields will be used to track the tumor in the operation. Thus accurate respiration motion evaluation would greatly reduce the radiation dose to the normal tissue and harm to the patient.

There are a number of methods have been proposed to deal with the organ motion estimation by registering point clouds or surfaces. According to the acquiring method of the point data, these methods can be summarized into two categories. One way is to use point data from feature extraction [12,13]. Castillo et al. [12] presented a framework for objective evaluation, and optical-flow method was used to register large sets of expert-determined landmark point pairs. Li et al. [13] presented a simulation method to exam the patient lung deformation induced by respiratory motion, and the lung deformation problem was solved by finite element analysis. Liu et al. [14] propose a shape-correlated statistical model on dense image deformations for patient-specific respiratory motion estimation, and a point-based particle optimization algorithm was used to obtain the shape models of lungs with group-wise surface correspondences. The other way is to use point data from surface segmentation [5,15–17]. Usually the lung surface is easy to be acquired using segmentation techniques because the hollow structure would lead to strong contrast intensity. Chui et al. [5] proposed the TPS–RPM (thin plate spline-robust point matching) algorithm, which formulated the non-rigid deformation by thin plate spline model, and EM-like (expectation maximization-like) method was used to obtain the final transformation parameters. Amberg [17] proposed the non-rigid ICP (iterative closest point) algorithm using a locally affine regularization, and the new regularization term assigned an affine transformation to each vertex and minimized the difference in the transformation of neighbouring vertices. Myronenko et al. [15] proposed the CPD (coherent point drift) method. The method imposed the coherence constraint by regularizing the displacement field, and the variational calculus was utilized to derive the optimal transformation using GMM (Gaussian mixture model) and EM.

In CPD and TPS-RPM, it is required that the source point cloud and the reference point cloud are in one-to-one corresponding relationship, and this condition is difficult to be satisfied in the practical application. Usually, the surface information is acquired through image segmentation techniques, and the corresponding information is lost during the procedure. Feature extraction techniques can obtain the point clouds that are in one-to-one corresponding relationship, but it cannot guarantee dense points on the lung surface. In addition, CPD and TPS-RPM utilize point-point distance to estimate the motion fields, and the registration may be trapped in local minima caused by sliding motion [1,2]. In this paper, we present a novel estimation method for lung motion based on the non-rigid registration of point clouds. The point clouds are segmented from 4DCT images and they represent the status of lung at different breathing phases. The proposed method formulates the distance term by tangent-plane distance, and it alleviates the requirement that the source point cloud and the target point should be in one-to-one corresponding relationship. Besides, the proposed method reduces the constraint of the motion along the tangent plane, and it improves the problem that the registration would be trapped in local minima caused by the sliding motion. Local affine transformation model is used to express the non-rigid deformation, and it solves the problem that the registration may deteriorate at the beginning of the optimization procedure using the EM method. The final objective function is expressed in the Frobenius norm formation, and a stochastic gradient descent strategy is used to find out the optimal transformation parameters.

In Section 2, a novel similarity metric for non-rigid point cloud registration using tangent-plane distance is presented and its implementation is elaborated. In Section 3, non-rigid registration experiments are carried out to validate the proposed method. The discussion and conclusion are presented in Section 4 and Section 5.

## Method

### The proposed similarity measure

Various algorithms exist for respiratory motion estimation based on non-rigid registration of point clouds, and they aim to recover the coordinate transformation required to align the source point cloud to the target point cloud. The estimation of respiratory motion fields between two point clouds $V_{i},\;V_{j}:\;X\subset {\mathbb{R}}^{3}\to {\mathbb{R}}^{3}$ can be formulated as searching for a transformation *X* between points of *V*_*i*_ and *V*_*j*_ by minimizing a distance/dissimilarity measure between the point clouds. Considering the ill-posed character inherent to non-rigid registration, an additional regularization term is introduced to provide particular smoothness properties of the transformation [2]. Usually the final objective function consists of two terms: the distance term and the regularization term, and the objective function can be formulated as follows

$$E\left(X\right)=E_{d}\left(X\right)+\alpha E_{s}\left(X\right)$$ (1)

where *E*_*d*_(*X*) and *E*_*s*_(*X*) represent the distance term and the regularization term respectively. The symbol *X* denotes the transformation parameters to be determined.

Former methods [15,17] usually formulate the distance term by point-point distance. The point-point distance is based on the assumption that one point in the source point cloud can map to a corresponding point in the target point cloud. The assumption requires some pre-operations, such as feature extraction or statistical shape model. However, it is difficult to implement these pre-operations to the non-rigid lung surface registration because they cannot provide dense points on the lung surface. The lung surface is usually segmented from 4DCT images, and during the segmentation procedure, the corresponding information is lost. Besides, many researchers indicate that the lung motion estimation suffers from the sliding motion [1,2,11]. A possible solution is to reduce the constraint of the motion along the tangent direction. In this paper, the tangent-plane distance is used to express the similarity between the two point clouds. The tangent-plane distance can be expressed as follows

$$E_{d}\left(X\right)={\left\|\left(\left(\left(w⊗I_{3}\right)\left(\left[\begin{array}{ccc} X_{1} & & \\ & \ddots & \\ & & X_{n} \end{array}\right]\left[\begin{array}{c} v_{1}\\ \vdots \\ v_{n} \end{array}\right]-\left[\begin{array}{c} u_{1}\\ \vdots \\ u_{n} \end{array}\right]\right)\right)\circ N\right)O\right\|}_{F}^{2}$$ (2)

where *X*_*i*_ is a 3×4 matrix, and the matrix denotes the local affine transformation. The local affine parameters are determined by minimizing the objective function. *w* = diag(*w*_1_,⋯*w*_*n*_) is the weight value, and the operator ⊗ means the Kronecker product. Symbol *v*_*i*_ represents the coordinates of a point in the source point cloud, and symbol *u*_*i*_ represents the coordinates corresponding point of *v*_*i*_ in the target point cloud. *N* is the normal vector of the target point, which can be calculated from the target point cloud. The operator ∘ represents the Hadamard product, and *O* = [1,1,1,1]^*T*^.

**Fig 1:**
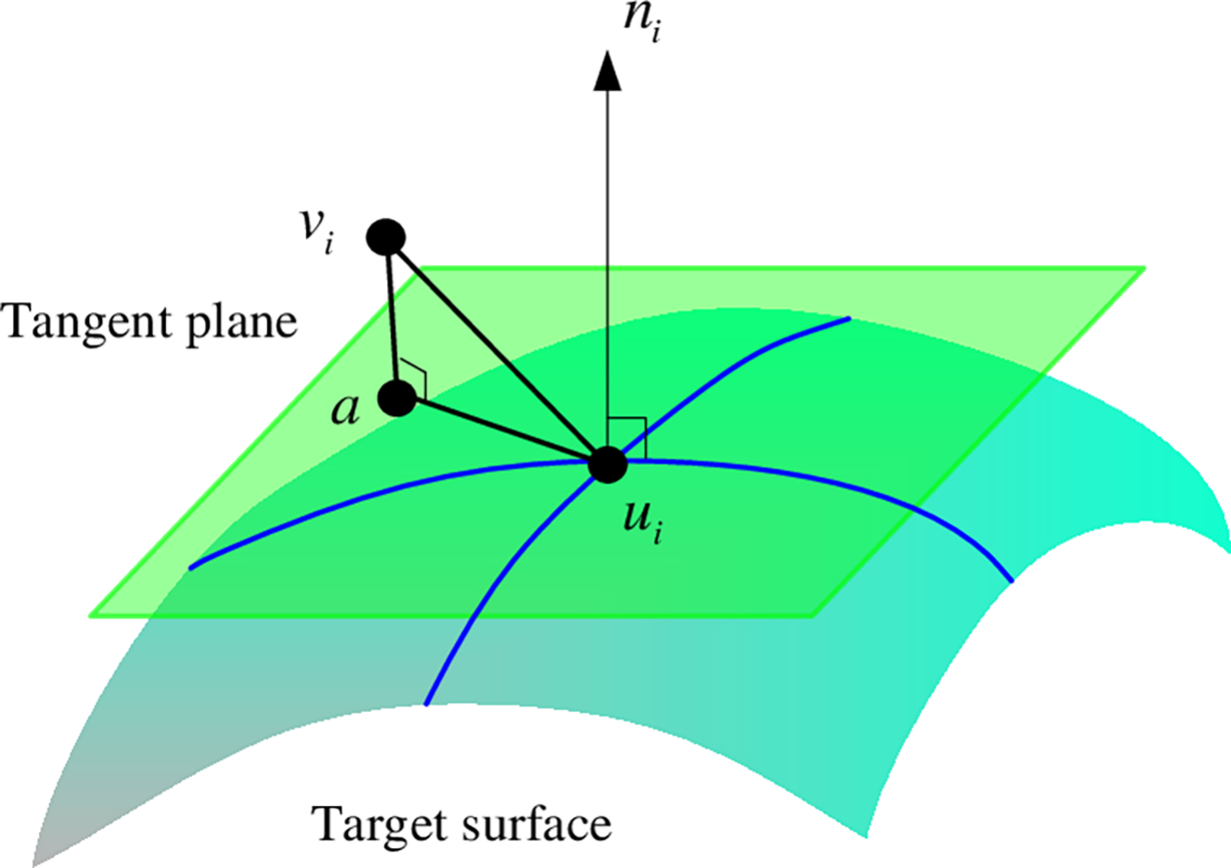
The illustration of the proposed method.

As shown in Fig 1, *v*_*i*_ is one point in the source point cloud, and *u*_*i*_ is its closest point in the target point cloud. The symbol *n*_*i*_ is the normal vector of *u*_*i*_. The distance term *E*_*d*_(*X*) represents the sum of squared tangent-plane distance, and the tangent-plane distance $\left|\vec{v_{i}a}\right|$ denotes the distance from point *v*_*i*_ to the tangent plane of point *u*_*i*_. In practical application of lung motion filed estimation, the interval of the point cloud is much smaller than the curvature radius of the target surface, and the tangent-plane distance is much smaller than the point-point distance. The proposed criterion reduces the constraint of the motion along the tangent direction. Since severe sliding motion exists in the lung region, the new criterion makes the residual registration error distribution more evenly and thus improves the lung motion estimation accuracy.

The regularization term can avoid both physically implausible displacement fields and local minima during optimization. In this paper, the regularization term is defined as the differences between the transformation matrices assigned to neighboring vertices [17]. The regularization term is expressed as follows

$$E_{s}\left(X\right)={\left\|\left(M⊗G\right)\left|\begin{array}{c} X_{1}\\ \vdots \\ X_{n} \end{array}\right|\right\|}_{F}^{2}$$ (3)

where *M* is the connectivity matrix. It is determined by the adjacent relation of the points in the source point cloud. The triangulation information of the source point cloud is used to compute the matrix. Point cloud Crust algorithm is used to generate the triangulation. *G* = *diag*(1,1,1,*γ*) is the stiffness matrix, and *γ* can be used to weight differences in the rotational and skew part of the deformation against the translational part.

In non-rigid ICP and CPD, the distance term is nonzero at the ideal transformation when the source point cloud and the target point cloud are in non-corresponding relationship, and this characteristics would make that the registration is more prone to get trapped in local minima. In our method, the distance term of the proposed method is close to zero at the ideal transformation whether the source point cloud and the target point cloud are in one-to-one corresponding relationship or non-corresponding relationship. Since the normal information is used in the proposed method, the constraint of the motion along the tangent direction is reduced, and it would benefit the registration of lung, where there exists large sliding motion. Furthermore, our method utilizes local affine transformation model to express the non-rigid deformation, and it could make the registration under small deformation circumstance and avoid the problem that the optimization may deteriorate at the beginning of the optimization procedure.

**Fig 2:**
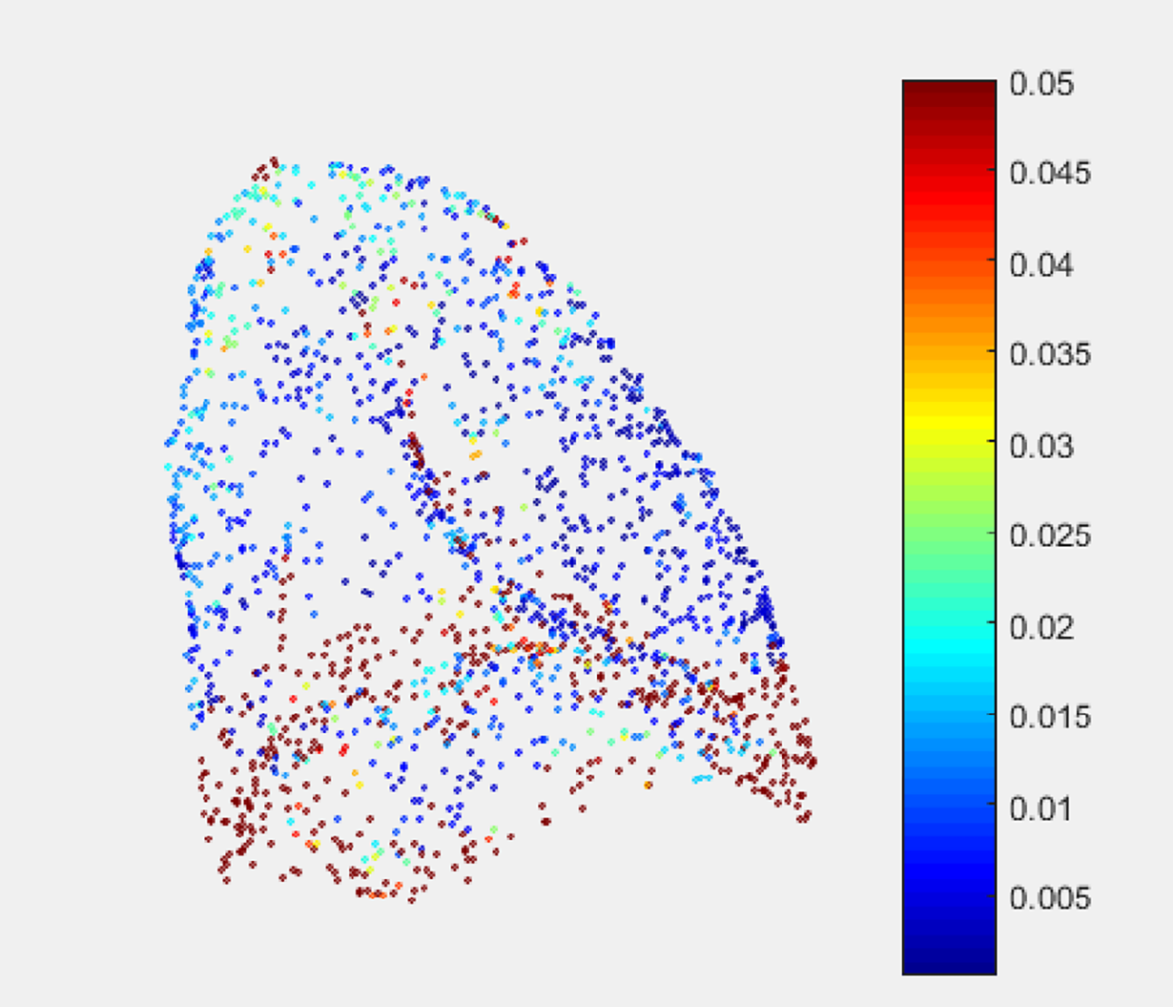
The illustration of surface variation of the lung.

Fig 2 illustrates the covariance analysis [18] of the left lung. The surface variation value means the structure sensitivity to the transformation. Small surface variation means that the local structure makes the distance term (typically ICP) irrelevant to one direction motion (usually the motion along the tangent plane), and big surface variation means that the distance term is sensitive to the transformation and suffers less from sliding motion. If the surface variation value is zero, it means the vicinity points lie in the plane at the point. In fact, the region of small surface variation is more prone to suffer from sliding motion. Because the distance term using non-rigid ICP is not zero, the point in this area is more prone to be trapped to the nearest point. However, the distance term using the proposed method is zero and the proposed criterion reduces the constraint of the motion along the tangent direction, then the regularization term can contribute to the registration.

### Implementation

Accurate respiratory motion estimation is important in radiation therapy of lung cancer. To acquire the lung motion fields, dense points are segmented from 4DCT images at different phases, then the dense point clouds are simplified to a relative small scale to reduce computation complexity. Finally, non-rigid point cloud registration using tangent-plane distance is carried out to establish the respiratory motion fields. A step-by-step protocol for the implementation is available on protocols.io (dx.doi.org/10.17504/protocols.io.qrhdv36). In this paper, local affine transformation model is used to express the non-rigid deformation. The non-rigid deformation of the point cloud is a combination of many local affine transformation, and each affine transformation matrix is assigned to each point in the point cloud. The local affine transformation *X*_*i*_ is a 3×4 matrix, and the total number of parameters to be determined is 12*n*, where *n* is the number of points in the source point cloud. To acquire the lung motion field, the two point clouds should be well registered and the transformation parameter *X* that minimizes the objective function should be found. In this paper, a stochastic gradient descent strategy is used to solve the optimization problem, and the derivative of the objective function to the transformation parameters is required during each iteration. According to the above paragraphs, the objective function can be expressed as follows

$$\begin{array}{l} E\left(X\right)=E_{d}\left(X\right)+\alpha E_{s}\left(X\right)\\ ={\left\|\left(w⊗I_{3}\right)\left(\left[\begin{array}{ccc} X_{1} & & \\ & \ddots & \\ & & X_{n} \end{array}\right]\left[\begin{array}{c} v_{1}\\ \vdots \\ v_{n} \end{array}\right]-\left[\begin{array}{c} u_{1}\\ \vdots \\ u_{n} \end{array}\right]\right)\right\|}_{F}^{2}+\alpha {\left\|\left(M⊗G\right)\left|\begin{array}{c} X_{1}\\ \vdots \\ X_{n} \end{array}\right|\right\|}_{F}^{2} \end{array}$$ (4)

To simplify the optimization procedure, it is required to convert the transformation parameters into the same formation, and after a matrix cracking the objective function can be expressed as follows

$$\begin{array}{l} E\left(X\right)=E_{d}\left(X\right)+\alpha E_{s}\left(X\right)\\ ={\left\|\left(\left(\left(w⊗I_{3}\right)\left(\left[\begin{array}{ccc} X_{1} & & \\ & \ddots & \\ & & X_{n} \end{array}\right]\left[\begin{array}{c} v_{1}\\ \vdots \\ v_{n} \end{array}\right]-\left[\begin{array}{c} u_{1}\\ \vdots \\ u_{n} \end{array}\right]\right)\right)\circ N\right)O\right\|}_{F}^{2}+\alpha {\left\|\left(M⊗G\right)\left|\begin{array}{c} X_{1}\\ \vdots \\ X_{n} \end{array}\right|\right\|}_{F}^{2}\\ ={\left\|\left(\left(W\left(\left[\begin{array}{ccc} v_{1}^{T} & & \\ & \ddots & \\ & & v_{n}^{T} \end{array}\right]\left|\begin{array}{c} X_{1}\\ \vdots \\ X_{n} \end{array}\right|-U\right)\right)\circ N\right)O\right\|}_{F}^{2}+\alpha {\left\|\left(M⊗G\right)\left|\begin{array}{c} X_{1}\\ \vdots \\ X_{n} \end{array}\right|\right\|}_{F}^{2}\\ =\alpha {\left\|\left(M⊗G\right)X\right\|}_{F}^{2}+{\left\|\left(\left(W\left(DX-U\right)\right)\circ N\right)O\right\|}_{F}^{2}\\ =\alpha {\left\|A_{1}X\right\|}_{F}^{2}+{\left\|\left(\left(A_{2}X-B_{2}\right)\circ N\right)O\right\|}_{F}^{2} \end{array}$$ (5)

Then the derivative of the objective function to the transformation parameters can be obtained using matrix derivative strategy.

$$\frac{dE\left(X\right)}{dX}=2\alpha A_{{}_{1}}^{T}A_{1}X+2A_{2}^{T}\left(N\circ \left(A_{2}X-B_{2}\right)\circ N\right)OO^{T}$$ (6)

In our method, the stochastic gradient descent strategy is used to obtain the new transformation parameters in each iteration, and the computation of the new variables is given as follows

$$X_{new}=X_{old}-\Delta L\frac{dE\left(X_{old}\right)}{dX_{old}}$$ (7)

where Δ*L* denotes the step length. Our method can be summarized in the following pseudo-codes.

- Initialize *X* and *M*.
- Initialize *γ* and *α*.
- Carry out the gradient descent optimization, and repeat until convergence (using Eq 7).
- Calculate the transformed source point cloud *Y* = *DX*.

## Experiments and results

The proposed method for respiratory motion estimation is validated by both artificial data experiments and real data experiments in this section. The experiments are conducted in the Matlab R2015b platform, which runs on Windows 7 operation system with Intel i5-2300 (2.80GHz) and 10G of RAM. Part of the code in the non-rigid ICP [17] is used in our experiments. The 4DCT images tested in the clinical data experiments are from popi-model database, in which the motion fields are provided by the contributors. The property would benefit the comparison of the different methods. In this paper, the registration error (in millimeters) is defined as mean registration error (MRE), and it can be defined as

$$\varepsilon =\frac{1}{n}\sum \left|{\vec{T}}_{true}-{\vec{T}}_{estimated}\right|$$ (8)

where ${\vec{T}}_{true}$ and ${\vec{T}}_{estimated}$ denote the motion vectors of the points in the true motion field and the estimated motion field, respectively, and *n* is the number of points in the source point cloud. The operator |•| means the magnitude of the vector.

### Artificial experiment

**Fig 3:**
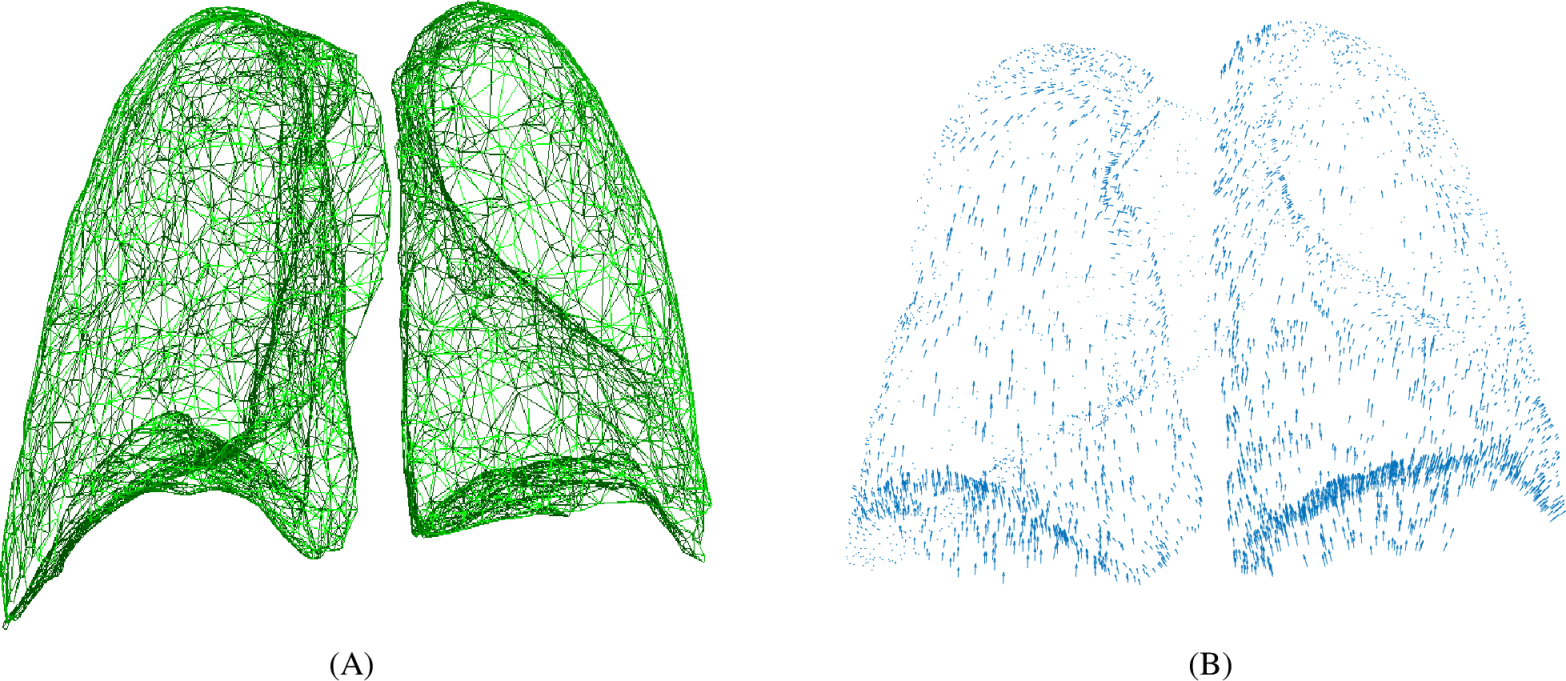
The experiment data in the artificial experiment. (A) The source point cloud. (B) The artificial motion field.

In this section, artificial data is used to implement the registration experiment. A synthetic motion field, which represents the deformation between the end-inspiration (IE) phase and the end-expiration (EE) phase, is used to generate the target point cloud. First, dense point cloud *F* is acquired by medical image segmentation and lung surface reconstruction, and dense point cloud *R* is generated by introducing the synthetic motion field into *F*. The point clouds *F* and *R* represent the status of lung surface at IE-phase and EE-phase, respectively. The surface normal vector *N* can be computed by *R*. Then *F* and *R* are simplified to *F*' and *R*' separately, and the surface normal vector *N* is also simplified to *N*' according to its correspondence with *R*. During the simplifying procedure, the corresponding information is lost. The point clouds *F*' and *R*' are not in one-to-one corresponding relationship, and a point in *F*' may not map to a corresponding point in *R*' exactly. Besides, usually the motion estimation of the left lung and the right lung can be conducted separately to reduce the computation complexity. The source point cloud is shown in Fig 3A, and the artificial motion field is shown in Fig 3B.

**Fig 4:**
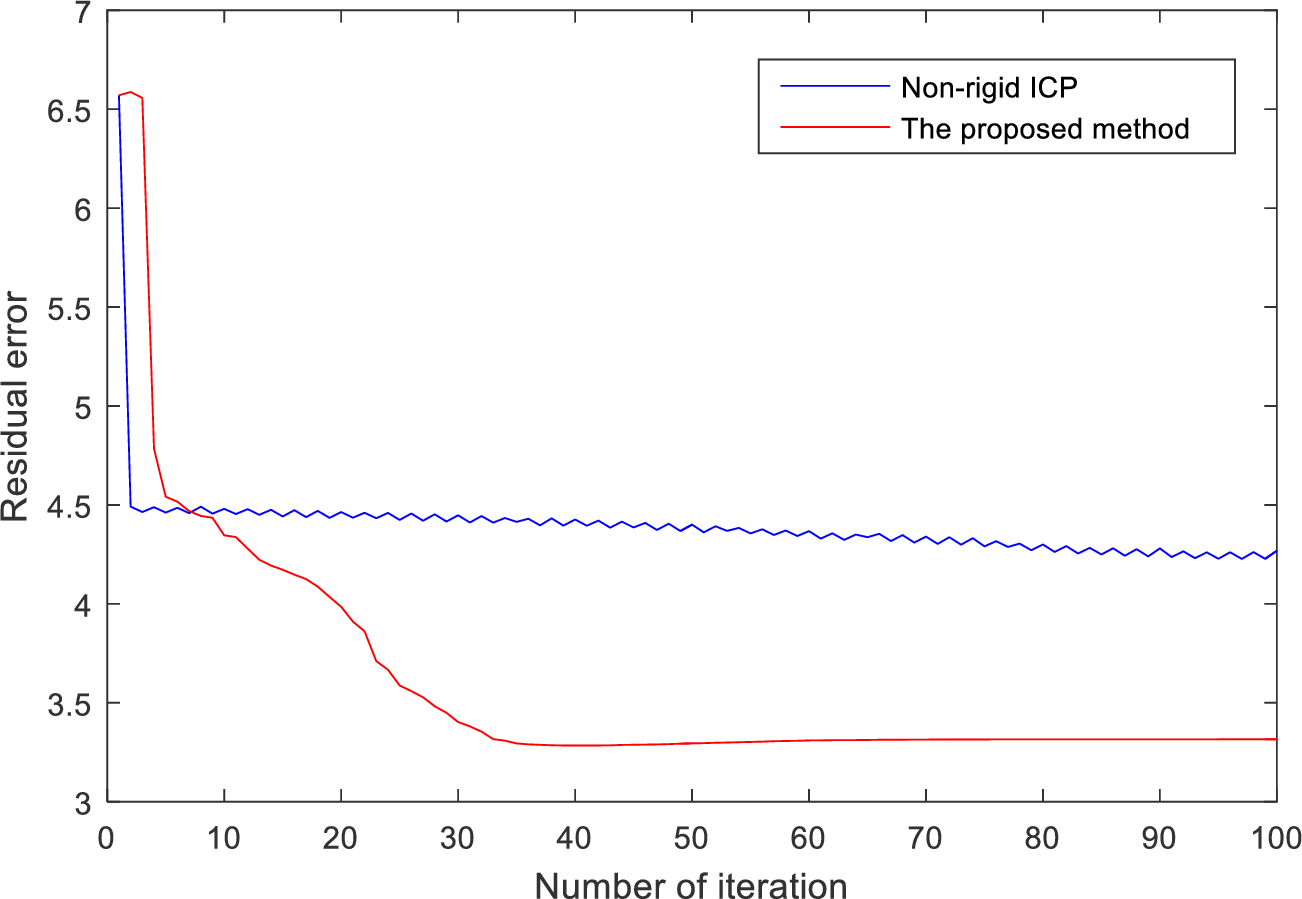
Registration error with respect to the number of iteration in the artificial experiment.

**Fig 5:**
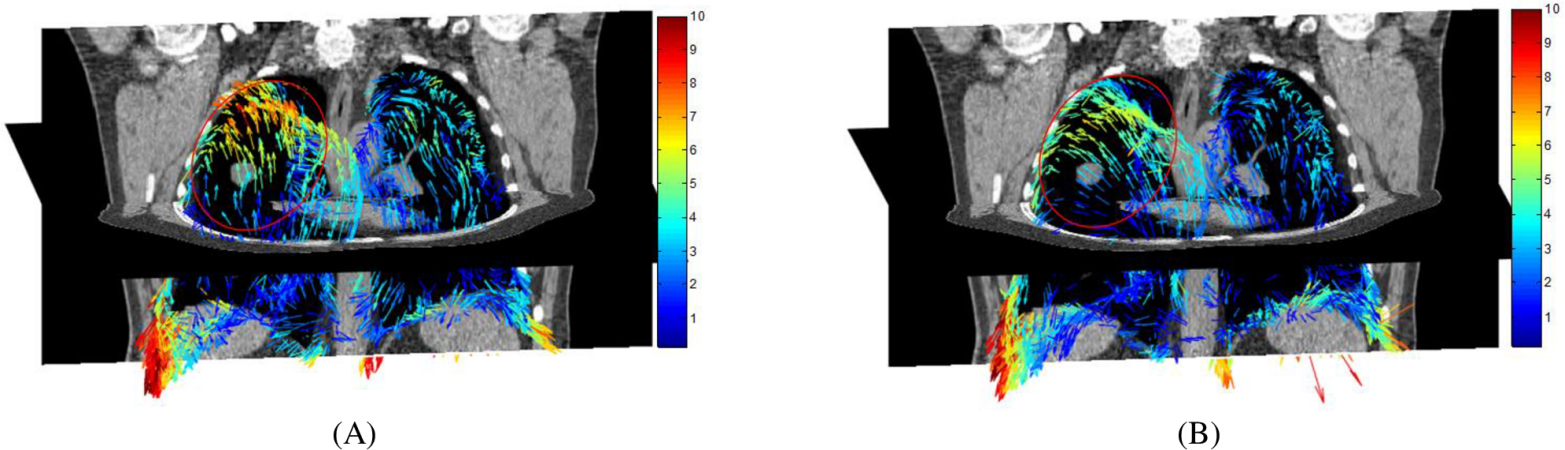
Registration error distribution using non-rigid ICP and the proposed method in the artificial experiment. (A) Registration error distribution using non-rigid ICP. (B) Registration error distribution using the proposed method.

In Fig 4, it can be observed that the final residual error using non-rigid ICP is 4.2687, and the final residual error using the proposed method is 3.3155. The proposed method achieves higher registration accuracy than non-rigid ICP. The reason is that non-rigid ICP sets up on the assumption that a point in the point cloud can map to a corresponding point in another point cloud. The distance term of non-rigid ICP is a non-zero value at the ideal transformation given that the interval of the point cloud is much smaller than the curvature radius of the target surface. The registration is prone to get trapped into local minima using non-rigid ICP. In practical application the one-to-one corresponding relationship is difficult to be satisfied. Usually the point clouds are segmented from 4DCT images and simplified into a relatively small scale. The corresponding relationship between the source point cloud and the target point cloud is unknown and the point in the source point cloud may not map to a corresponding point in the target point cloud. In addition, the residual error using non-rigid ICP reduces much faster than that the proposed method. The registration result using non-rigid method becomes stable after 6 iterations, and the registration result using the proposed method become stable after 40 iterations. The reason is that non-rigid ICP provides an analytical solution during each iteration, while the proposed method utilizes a stochastic gradient descent strategy and the gradient of the objective function with respect to the transformation parameters is used to obtain the new transformation parameters in each iteration. In Fig 5, it can be observed that the new method outperforms non-rigid ICP. As shown in Fig 3B, the motion is caused by the bottom diaphragmatic muscle, and there exists severe sling motion in the lateral areas of the lung. In Fig 5, the residual errors are mainly along the tangent plane. However, the magnitude of the residual error using the proposed method is much smaller than that using non-rigid ICP. The reason is that the distance term of the proposed method is zero when the source point cloud gets close to the target point cloud. The proposed method reduces the constraint of the motion along the tangent plane and the regularization term would contribute to the registration. There remain large registration errors towards the bottom of the lung. using both the proposed method and non-rigid ICP. The reason may be that the breathing motion is arisen from the contraction of the diaphragmatic muscle area. The magnitude of the motion on the lower lobe of the lung is relatively large and large magnitude of the motion on the lower lobe of the lung will result large registration error.

### 4DCT data experiment

In this section, clinical 4DCT images from popi-model [4] are used to conduct the registration experiment. The reference motion fields are provided by the providers of the dataset. The providers of the popi-model implement the Demons algorithm to deformably register the reference phase to all other volumes. The method utilizes the whole intensity information of the 4DCT images and it takes hours to obtain the motion field, and it makes the provided motion fields quite accurate. The characteristics would benefit the comparison of the different methods. The proposed method is compared with non-rigid ICP and CPD. First, dense point clouds *F* and *R* at different phases are segmented separately using threshold segmentation method, and 3DMed software is used to conduct the procedure. Then *F* and *R* are simplified to *F*' and *R*' by curvature sampling method and stochastic sampling method, and Geomagic software is used in the procedure. The final point clouds to be registered are in non-corresponding relationship, which means that one point in the source point cloud cannot map to one point in the target point cloud. Non-rigid registration experiments with five different 4DCT image pairs are carried out in this section. For each image pair, experiments with different numbers of points in *F*' are conducted to reduce the influence of contingency. The number of points in *F*' is set as 3600, 3800, 4000, 4200 and 4500, and the number of points in *R*' is 8000. Non-rigid registrations experiments using CPD are conducted with the number of points in *F*' set as 4000. The numbers of points in *F*' and *R*' are different in this section. The complexity of the optimization procedure is directly related to the scale of *F*' because the scale of *F*' determines the number of the deformation parameters. Large scale of *R*' can reduce the sampling error and interpolation error during the optimization procedure. So the scale of *F*' is relatively small and the scale of *R*' is relatively large.

**Fig 6:**
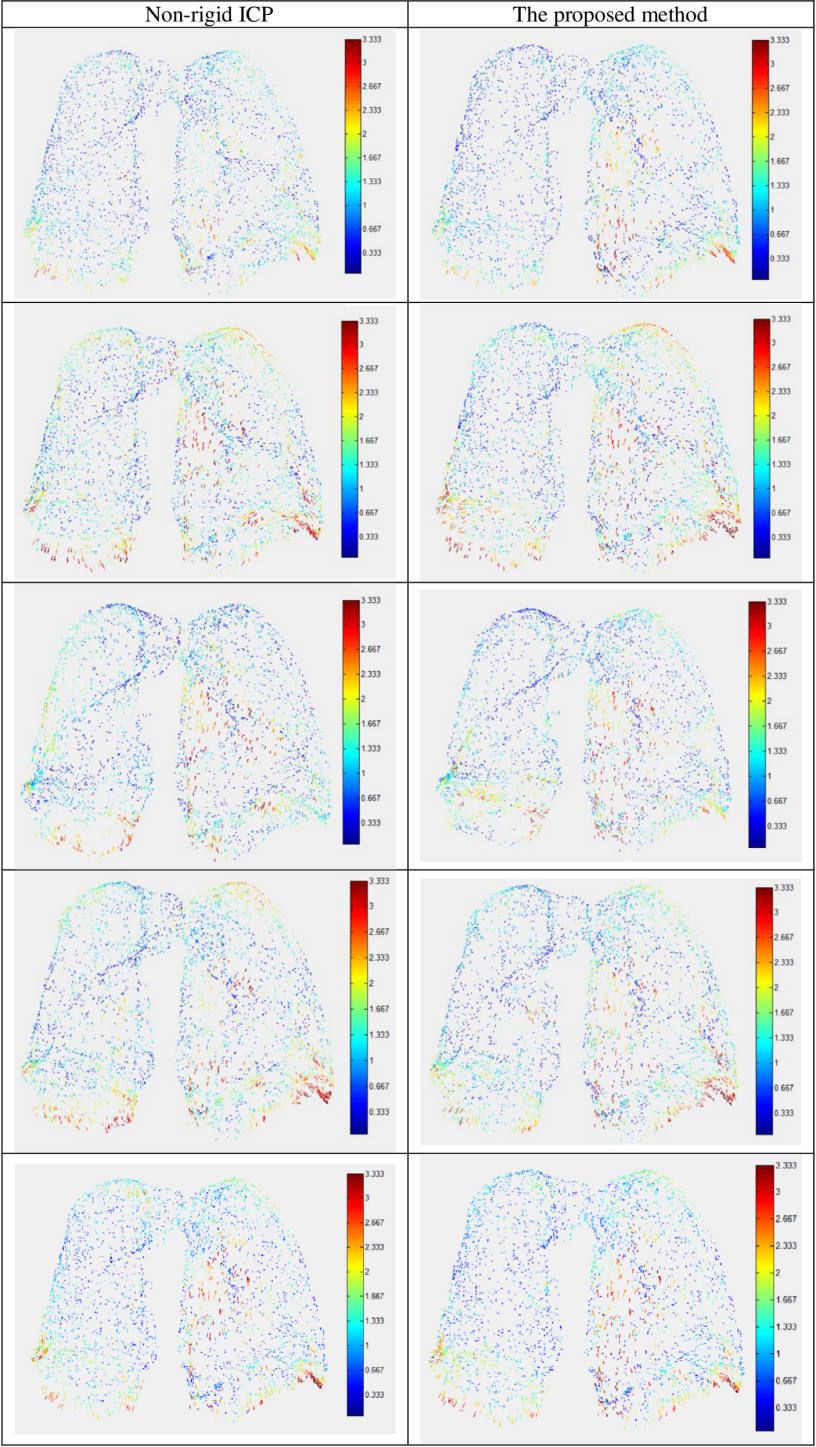
Registration error distribution using non-rigid ICP and the proposed method when the number of points in *F*' is set as 4000 in the 4DCT data experiments.

The registration results are shown in Table 1. It can be observed that, the proposed method performs best among the three methods tested and it achieves a better registration accuracy in every experiment. When the number of points in *F*' is 4000, our method outperforms non-rigid ICP with about 10%~30% accuracy increase. CPD performs much worse than both the proposed method and non-rigid ICP. The reason is that CPD formulates the registration problem using GMM and the transformation parameters are solved the by EM method. This strategy leads to that the target point would shrink at the beginning of the optimization procedure and then expand to the target point cloud. For the initial iterations, the distance between the source point cloud and the target point cloud increases, and the transformation deviates from the ideal status. In our method, the local affine transformation model is used to represent the transformation, and the deformation of the source point cloud is controlled under a small range, thus the proposed method solves the problem that the registration may deteriorate at the beginning of the optimization procedure. The registration error distribution maps of non-rigid ICP and the proposed method when the number of points in *F*' is set as 4000 are shown in Fig 6, where the proposed method achieves much better accuracy than non-rigid ICP. Comparing the error distribution of the two methods, it can be observed that the proposed method can improve the sliding problem. The reason is that the distance term using the proposed method is zero value at the ideal transformation. It can reduce the constraint of the motion along the tangent direction, thus it can improve the problem that the registration is prone to be trapped into local minima caused by sliding motion.

**Table 1:** The registration errors (in millimeters) using CPD, non-rigid ICP and the proposed method in the 4DCT data experiment.

Method	Non-rigid ICP					The proposed method					CPD
Number of points in *F*'	3600	3800	4000	4200	4500	3600	3800	4000	4200	4500	4000
Image pair 1	0.9664	1.1147	0.9806	1.0053	0.9523	**0.7963**	**0.8022**	**0.7954**	**0.8080**	**0.7923**	2.1970
Image pair 2	1.3879	1.4936	1.3845	1.3869	1.3090	**1.2639**	**1.2160**	**1.2661**	**1.2468**	**1.2014**	2.7650
Image pair 3	1.3278	1.3361	1.2096	1.2042	1.1091	**1.0514**	**1.1362**	**0.9887**	**0.9972**	**0.9836**	2.5994
Image pair 4	1.4972	1.3681	1.4095	1.3577	1.3082	**1.1924**	**1.1451**	**1.1052**	**1.0452**	**1.0218**	2.5362
Image pair 5	1.1874	1.3898	1.1591	1.1270	1.2134	**1.0324**	**1.0975**	**0.9857**	**1.0785**	**0.9469**	2.4251

### Artificial experiment with corresponding relationship

**Fig 7:**
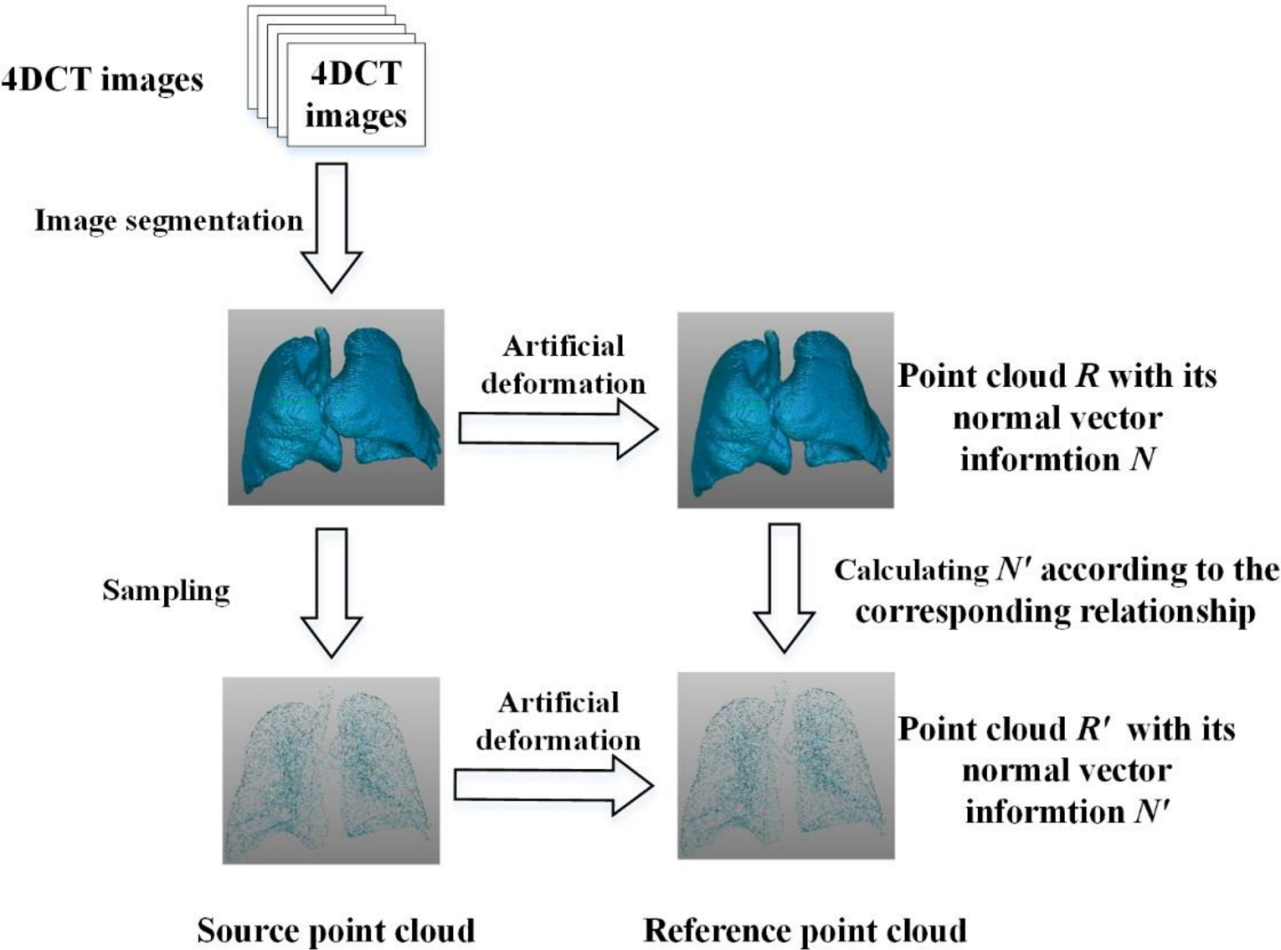
Illustration of the flowchart in the artificial experiment with corresponding relationship.

In this section, artificial experiments with corresponding relationship are conducted to acquire the motion field of the left lung. The point clouds are in one-to-one corresponding relationship. In each experiment, a synthetic motion field between different phases is utilized to generate the point clouds. Fig 7 illustrates the flowchart in the experiment. First, a dense point cloud *F* is acquired by medical image segmentation and lung surface reconstruction. Then the point cloud *R* is obtained by introducing the artificial motion field to the point cloud *F*, and *R* represents the target surface. Usually, the point cloud *F* and *R* have a relatively large scale (over 20,000), and it would make the optimization problem too complicated. The point cloud *F* is simplified to *F*' by the curvature sampling method and stochastic sampling method. The simplified point cloud *R*' is acquired by introducing the artificial motion field to point cloud *F*'. Thus the point clouds *F*' and *R*' are in one-to-one corresponding relationship, which means that each point in *F*' can find a corresponding point in *R*'. According to the definition of the proposed method, the normal vector information *N*' is needed in the implementation. The surface normal vector information *N* can be computed by *R*, and *N*' can be acquired according to the corresponding relationship between *F* and *R*. *N*' is not directly calculated from *R*' because dense point cloud in *R* can provides more accurate normal information. Then non-rigid registration between *F*' and *R*' is conducted to establish the respiratory motion field.

**Fig 8:**
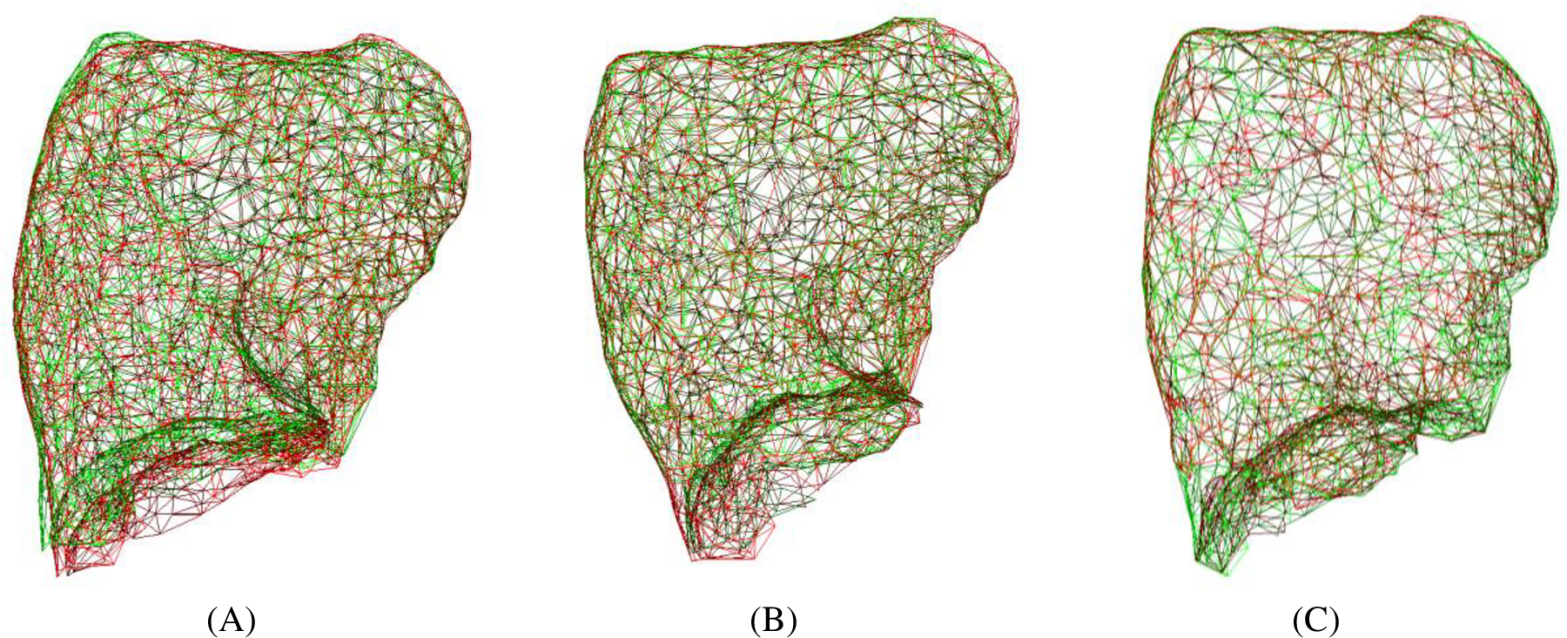
The point clouds before and after registration in the artificial experiments with corresponding relationship. (A) Overlapped left lung before registration. (B) Overlapped left lung after registration using the proposed method. (C) Overlapped left lung after registration using non-rigid ICP.

Three non-rigid registration experiments are conducted in this section. The point clouds and the results using the proposed method and non-rigid ICP in the first experiment is shown in Fig 8. Table 2 shows the residual registration errors using non-rigid ICP and the proposed method for the three experiments. It can be observed that the proposed method achieves the similar registration accuracy with non-rigid ICP for all the experiments conducted. Comparing with the performance of the two methods in the first experiment, it can be observed the main difference is that in this section the source point cloud and the target point cloud are in one-to-one corresponding relationship, which means that a point in the source point cloud can map to one corresponding point in the target point cloud. The corresponding relationship would make the distance term of non-rigid ICP is zero value at the ideal transformation, and it would improve the registration accuracy of the non-rigid ICP method. The different performance between the first experiment and this section indicates that the proposed method does not require the assumption that the source point cloud and the target point cloud are in one-to-one corresponding relationship. The assumption is difficult to be satisfied in practical respiratory motion estimation using non-rigid lung surface registration.

**Table 2:** The registration errors (in millimeters) using non-rigid ICP and the proposed method in the artificial experiments with corresponding relationship.

Example	Non-rigid ICP	The proposed method
1	1.5021	1.4664
2	2.3051	2.2974
3	1.8602	1.8569

## Discussions

Respiratory motion estimation is important in medical image processing. It is an essential procedure for many medical applications, such as image-guided interventions, quantitative evaluation of the motion and generating dynamic numerical phantom data for assessment. Usually thorax 4DCT images of the patient are acquired before radiation treatment of lung cancer. Then lung motion fields are calculated using 4DCT images. The motion fields will be used to track the tumor in the operation.

In this paper, a non-rigid point cloud registration based lung motion estimation method is proposed. Dense point clouds are segmented from 4DCT images at different phase. Then the dense point clouds are simplified to a relatively small scale to reduce computation complexity. Non-rigid registration of the point clouds is conducted to establish the lung motion fields. The proposed method employs the tangent-plane distance to represent the distance term. It can improve the problem that the registration is prone to be trapped into local minima caused by sliding motion when using non-rigid ICP. Local affine transformation model is used to express the non-rigid deformation of the lung motion, and the deformation of the source point cloud is controlled under a small range, thus the proposed method solves the problem that the registration may deteriorate in the initial iterations using CPD.

Before the non-rigid point cloud registration, a 4DCT image segmentation procedure and a point cloud simplifying procedure are conducted. The performance of image segmentation may be influenced by the existence of artifacts, tutors and data incompleteness. In this paper, the curvature sampling method and random point cloud are used during the simplifying procedure, and the influence of distribution of the simplified point cloud on non-rigid registration should be studied in future work. Moreover, in clinical applications, the surface motion cannot fully depict the motion of the chest. However, breathing motion is arisen from the contraction of the diaphragmatic muscle area. Once the lung surface motion is determined, the motion of the whole chest can be evaluated by techniques like finite element analysis and statistical shape model.

Non-rigid registration experiments are carried out to validate the proposed method. In 4DCT data experiment, the proposed method outperforms non-rigid ICP with about 10%~30% accuracy increase. The tangent-plane distance can reduce the constraint of the motion along the tangent direction. While the distance term of non-rigid ICP is a non-zero value at the ideal transformation given that the interval of the point cloud is much smaller than the curvature radius of the target surface, and the registration is prone to get trapped into local minima using non-rigid ICP. The influence of the one-to-one corresponding relationship is discussed in the experiments. The performance of the proposed method and non-rigid ICP is quite different in the registration experiment without one-to-one corresponding relationship and the registration experiment with one-to-one corresponding relationship. The phenomenon indicates that the proposed method does not require the assumption that the source point cloud and the target point cloud are in one-to-one corresponding relationship. The assumption is difficult to be satisfied in practical application.

## Conclusion

A novel method based on the non-rigid registration of the point clouds is proposed for lung motion estimation. The point clouds are segmented from 4DCT images and they represent the statuses at different phases. The proposed method employs the tangent-plane distance to represent the distance term, and local affine transformation model is used to express the non-rigid deformation of the lung motion. The final objective function is expressed in the Frobenius norm formation, and a stochastic gradient descent strategy is utilized to obtain the optimal local affine transformation parameters. The proposed method alleviates the requirement that the source point cloud and the target point cloud should be in one-to-one corresponding relationship. According to our definition, the proposed method reduces the constraint on the motion along the tangent plane.
